# Targeting the LY6H-PI3K/AKT autophagy axis suppresses HCC malignancy and reveals a druggable vulnerability

**DOI:** 10.1038/s41419-026-08947-z

**Published:** 2026-06-15

**Authors:** Fei Wei, Jie Wang, Ye Cheng, Caiyan Chen, Yong Wu, Jie Pan, Jiahong Wang, Yufei Wang, Meifeng Wang, Xueni Zeng, Aimin Huang

**Affiliations:** 1 Department of Pathology, School of Basic Medical Sciences, Fujian Medical University, Fuzhou, Fujian China; 2 Institute of Oncology, Fujian Medical University, Fuzhou, Fujian China

**Keywords:** Liver cancer, Liver cancer

## Abstract

Hepatocellular carcinoma (HCC) is a highly lethal malignancy worldwide, whose initiation and progression are closely associated with dysregulated autophagy. Lymphocyte antigen 6 family member H (LY6H), a glycosylphosphatidylinositol-anchored protein, is aberrantly expressed in multiple cancers; however, its functions and mechanisms in HCC remain undefined. Here, we demonstrate that LY6H is markedly upregulated in HCC specimens and that elevated LY6H expression correlates with poorer patient survival. Transcriptomic analyses link LY6H expression to enhanced autophagic activity in HCC cells. Mechanistically, LY6H directly binds the p85 subunit of PI3K, stabilizes its phosphorylation at Tyr467, and protects phosphorylated p85 from degradation, thereby promoting activation of the PI3K/AKT signaling pathway and driving autophagy. Functional assays in vitro, along with in vivo experiments utilizing the PI3K inhibitor LY294002, confirm that LY6H promotes HCC cell proliferation through a mechanism involving the PI3K/AKT pathway and autophagy. Importantly, we identify NSC243928 as a potential small-molecule inhibitor of LY6H, which effectively abrogates LY6H-driven autophagy and tumor-promoting functions both in vitro and in vivo. Immunohistochemical analyses reveal positive correlations among LY6H, ATG3, Beclin1, PI3K, and AKT expression in HCC tissues, and their co-overexpression predicts an adverse prognosis. Collectively, this work uncovers a critical regulatory role of the LY6H–p-PI3K–autophagy axis in HCC progression, elucidates the molecular mechanisms underlying LY6H-mediated oncogenic effects, and identifies NSC243928 as a promising therapeutic candidate for targeting LY6H in HCC.

## Introduction

Hepatocellular carcinoma (HCC) ranks as the third leading cause of cancer-related mortality worldwide, with incidence rising in certain regions and a five-year survival rate below 20% [1, 2]. Despite advances in therapy, outcomes remain poor due to tumor heterogeneity and resistance [3–6]. Autophagy—a fundamental metabolic process—exerts tumor-suppressive effects during early carcinogenesis but switches to support tumor survival and progression at advanced stages [7, 8]. Thus, elucidating the molecular control of autophagy in HCC is essential for devising novel treatment strategies.

Lymphocyte antigen 6 family member H (LY6H), an LY6/uPAR superfamily protein, has garnered attention because LY6 members are often dysregulated in malignancies and influence tumorigenesis through cell-cell interactions and signal transduction [9–14]. Our preliminary data show that LY6H is markedly overexpressed in HCC and correlates with poor prognosis. However, the mechanisms by which LY6H drives malignant progression remain undefined. Notably, transcriptomic analysis following LY6H knockdown revealed significant alterations in autophagy-related gene expression, suggesting that LY6H may promote HCC progression by modulating autophagic pathways.

The PI3K/AKT signaling cascade—a key regulator of autophagy—is aberrantly activated in roughly 50% of HCC cases, driving malignancy by enhancing proliferation and suppressing apoptosis [15–17]. Moreover, PI3K/AKT activation markedly amplifies autophagic flux in HCC cells, promoting their survival under stress [18]. Consequently, disrupting PI3K/AKT-mediated autophagy has emerged as a promising therapeutic strategy.

Building on our earlier work, we investigated whether LY6H modulates autophagy via PI3K/AKT to influence HCC progression. Integrating clinical samples with in vitro and in vivo studies, we elucidated a critical role for the LY6H–p-PI3K axis in regulating HCC autophagy. Using interaction and functional assays, we demonstrated that LY6H directly binds to the PI3K p85 subunit, stabilizes its phosphorylation at Tyr467, and protects phospho-p85 from degradation, thereby enhancing PI3K/AKT activity and autophagic flux. Importantly, we identified NSC243928 as a small-molecule LY6H inhibitor that attenuates LY6H-driven autophagy and tumor promotion in vitro and in vivo. Together, these findings clarify LY6H’s role in autophagy and provide a rationale for LY6H-targeted therapy in HCC.

## Results

### LY6H is highly expressed in HCC and negatively correlated with prognosis

**Fig. 1:**
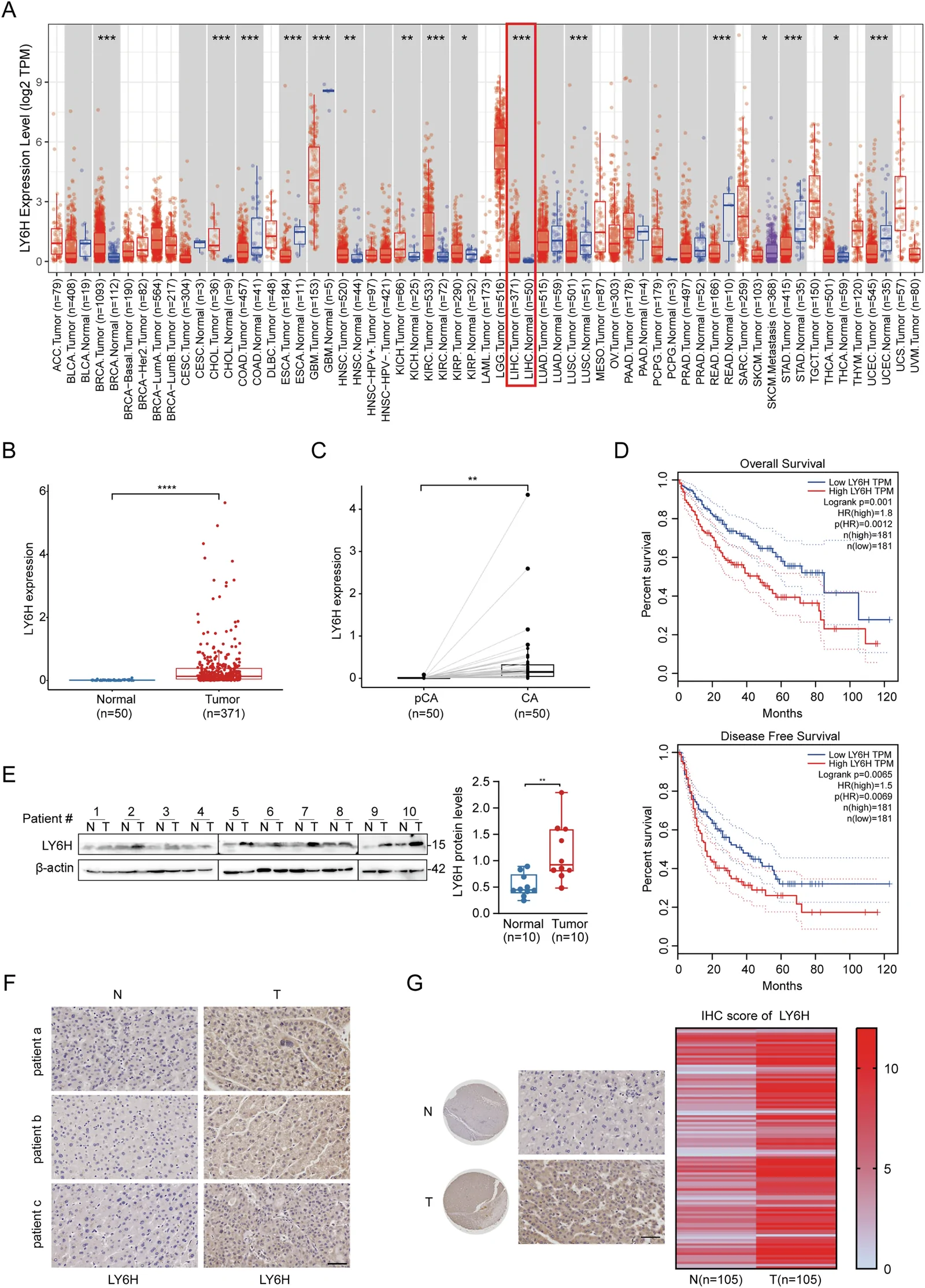
LY6H is highly expressed in HCC and negatively correlated with prognosis. **A** Expression profile of LY6H across 33 tumor types and corresponding normal tissues from the TIMER database. **B**, **C** LY6H expression status in the TCGA-LIHC dataset and across tumor versus paired adjacent normal tissues. **D** Kaplan–Meier survival analysis (log-rank test) showing the association between LY6H expression levels and patient prognosis (OS and DFS) in TCGA-LIHC (high LY6H expression group, *n* = 181; low expression group, *n* = 181). **E** Western blotting analysis of LY6H protein expression in 10 paired HCC and adjacent non-tumor tissues. **F** Representative IHC images showing LY6H expression in normal and tumor tissues in three cases (400× magnification; scale bar: 50 μm). **G** Representative images of LY6H tissue microarray and statistical results of immunohistochemical scores (normal tissues, *n* = 105; tumor tissues, *n* = 105). Data were displayed as *mean* *±* *SD*. (**p < 0.01; ****p < 0.0001).

To assess the differential expression of LY6H in tumor tissues, we examined its expression profile using the TIMER database, which integrates data from The Cancer Genome Atlas (TCGA). The analysis revealed that LY6H was significantly overexpressed in several tumor types, including liver hepatocellular carcinoma (LIHC), breast invasive carcinoma (BRCA), cholangiocarcinoma (CHOL), and kidney renal clear cell carcinoma (KIRC) (Fig. 1A). In contrast, LY6H expression was downregulated in bladder urothelial carcinoma (BLCA), esophageal carcinoma (ESCA), rectum adenocarcinoma (READ), and uterine corpus endometrial carcinoma (UCEC) (Fig. 1A). These findings suggest that LY6H may play distinct roles in different tumor types.

Focusing on liver cancer, LY6H expression profiling in TCGA-LIHC dataset and its subsequent paired analysis demonstrated significantly higher levels in liver cancer tissues compared to adjacent normal tissues (Fig. 1B, C). Survival analysis further indicated that elevated LY6H expression was associated with poor overall survival (OS) (log-rank *p* = 0.001) and disease-free survival (DFS) (log-rank *p* = 0.0065) in liver cancer patients (Fig. 1D). To validate these findings, we analyzed 10 paired HCC/adjacent tissue specimens by Western blotting, confirming increased LY6H protein levels in tumor tissues (Fig. 1E). Additionally, immunohistochemical (IHC) staining of three cases and a tissue microarray (TMA) containing 105 HCC specimens further corroborated LY6H overexpression in HCC tissues (Fig. 1F, G). These data suggest that LY6H may play a significant role in HCC pathogenesis and have clinical implications for patient prognosis.

### LY6H promotes tumor cell autophagy in HCC

**Fig. 2:**
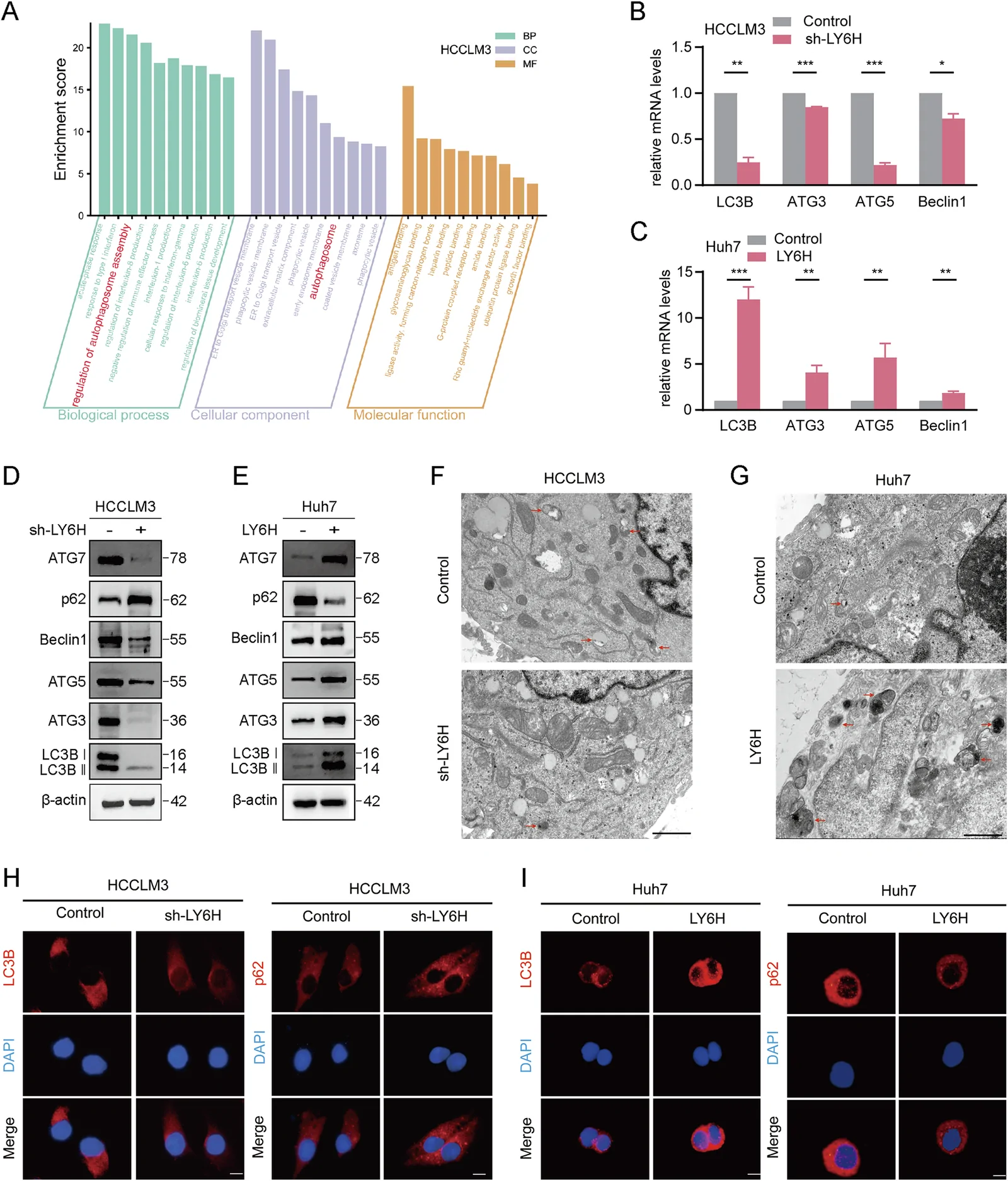
LY6H promotes tumor cell autophagy in HCC. **A** GO functional enrichment analysis of DEGs in HCCLM3 cells with LY6H knockdown compared to control cells. **B**, **C** qRT-PCR analysis of ATG3, ATG5, Beclin1, and LC3B expression levels in LY6H knockdown groups versus control groups in HCCLM3 cells, and in LY6H overexpression groups versus control groups in Huh7 cells, with 18S as the internal control. **D**, **E** Western blotting analysis of ATG3, ATG5, ATG7, Beclin1, LC3B-II, and p62 expression levels in LY6H knockdown groups versus control groups in HCCLM3 cells, and in LY6H overexpression groups versus control groups in Huh7 cells, with β-actin as the internal control. **F**, **G** Transmission electron microscopy (TEM) imaging of autophagosomes in HCCLM3 cells with LY6H knockdown versus control cells, and in Huh7 cells with LY6H overexpression versus control cells (red arrows indicate autophagosomes). Scale bar: 1 μm. **H**, **I** Immunofluorescence (IF) detection of LC3B and p62 in HCCLM3 cells with LY6H knockdown versus control cells, and in Huh7 cells with LY6H overexpression versus control cells. Red spots represent LC3B or p62, and blue spots represent DAPI. Scale bar: 50 μm. Data were displayed as *mean* *±* *SD*. (*p < 0.05; **p < 0.01; ***p < 0.001).

To investigate the functional role and underlying molecular mechanisms of LY6H in HCC, we first assessed LY6H expression in HCC cell lines (Fig. S1A, B). We then established stable LY6H-knockdown cell lines (LV-shLY6H) in both HepG2 and HCCLM3 cells through lentiviral transduction, with corresponding control cell lines generated in parallel (Fig. S1C, D). Transcriptome analysis via RNA sequencing identified 363 and 119 differentially expressed genes (DEGs) (|log₂FC | ≥ 1, *p* < 0.05) in HepG2 (Fig. S2A) and HCCLM3 (Fig. S2B) cells (see Supplementary Table 5), respectively, compared to control cells. Gene Ontology (GO) enrichment analysis of these DEGs revealed significant enrichment of genes involved in the regulation of autophagosome assembly within the biological processes category for both cell lines (results for HepG2 cells shown in Fig. S2C; results for HCCLM3 cells shown in Fig. 2A). At the cellular component level, DEGs specific to HCCLM3 cells were significantly enriched in modules related to autophagosome structure (Fig. 2A).

To functionally validate these findings, we performed comprehensive cellular experiments. Real-Time Quantitative Polymerase Chain Reaction (qRT-PCR) (Fig. 2B, S3A) and Western blotting (Fig. 2D, S3C) analyses showed that LY6H knockdown significantly decreased the expression of key autophagy-related genes (ATG3, ATG5, Beclin1, and LC3B-II) while increasing p62 expression. Transmission electron microscopy (TEM) revealed a marked reduction in autophagosome formation in HCC cells with LY6H depletion (Fig. 2F, S3E). Immunofluorescence (IF) microscopy further showed a decrease in LC3B puncta formation and an increase in p62 accumulation upon LY6H silencing (Fig. 2H, S3G). For reciprocal validation, we established LY6H-overexpressing SNU-449 and Huh7 cell lines (Fig. S1E, F), which conversely showed upregulated autophagy markers and augmented autophagosome production (Fig. 2C, E, G, I, S3B, S3D, S3F and S3H). Collectively, these data suggest that LY6H plays a critical role in regulating autophagic activity in HCC cells.

### LY6H activates the PI3K/AKT pathway to promote autophagy in HCC cells

To elucidate the molecular mechanisms by which LY6H regulates autophagy in HCC cells, we performed KEGG pathway enrichment analysis (*p* < 0.05) using RNA-Seq data to identify LY6H-related signaling pathways. The results demonstrated significant enrichment of the PI3K/AKT signaling pathway in both HepG2 (Fig. S4A) and HCCLM3 (Fig. S4B) cells. This pathway, a well-established regulator of autophagy, plays a crucial role in HCC progression [19, 20]_._ Further analysis of TCGA data revealed a positive correlation between LY6H expression and the key genes of the PI3K/AKT pathway (PIK3R6, PIK3CD, AKT1S1, and LAMTOR1) (Fig. S4C), suggesting that LY6H may influence autophagy through this pathway.

**Fig. 3:**
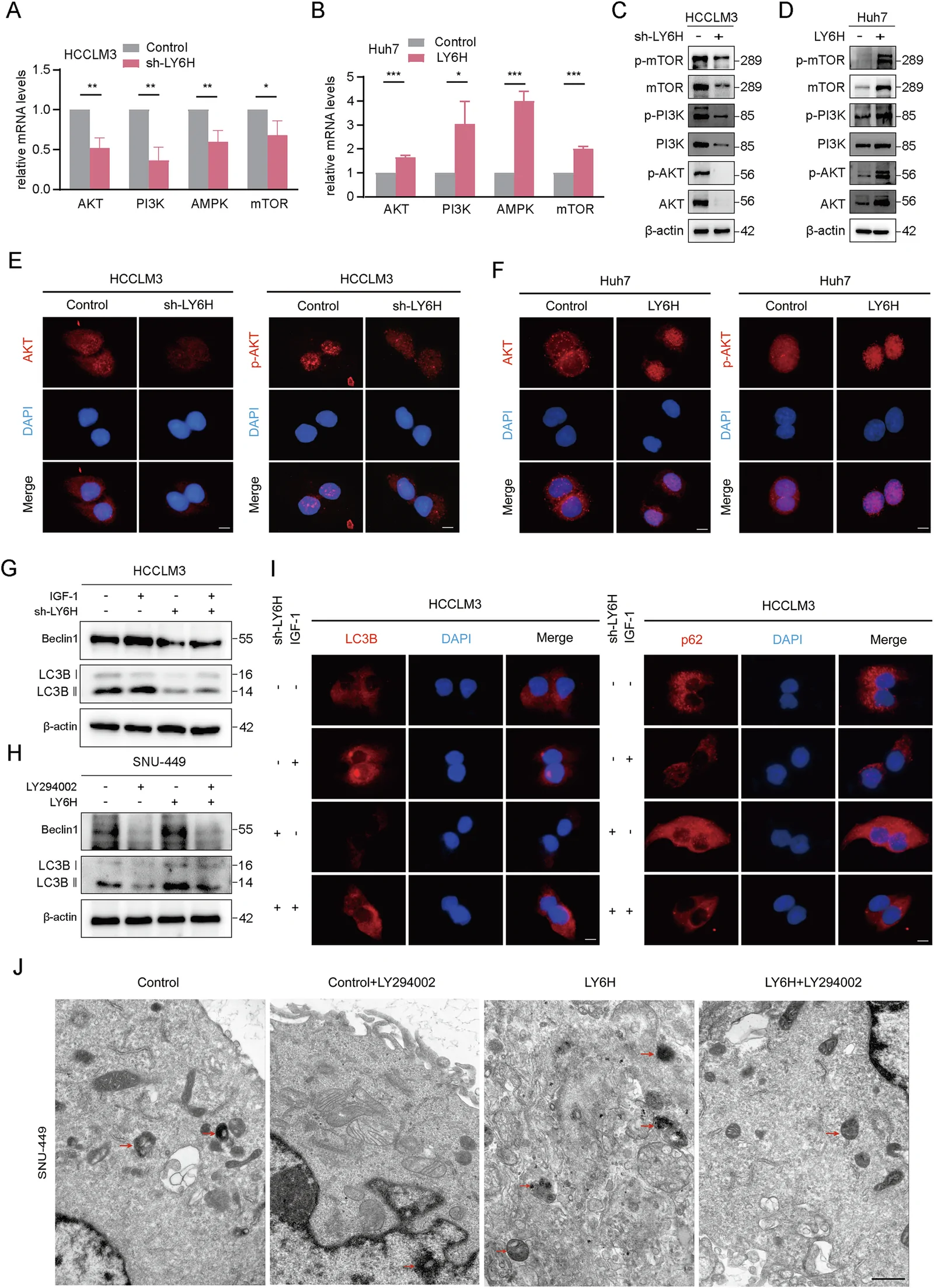
LY6H activates the PI3K/AKT pathway to promote autophagy in HCC cells. **A,** **B** qRT-PCR analysis of PI3K, AKT, AMPK and mTOR mRNA expression levels in LY6H knockdown groups compared to control groups in HCCLM3 cells, and in LY6H overexpression groups compared to control groups in Huh7 cells, with 18S as the internal control. **C**, **D** Western blotting analysis of AKT, PI3K, mTOR, and their phosphorylated forms in LY6H knockdown groups compared to control groups in HCCLM3 cells, and in LY6H overexpression groups compared to control groups in Huh7 cells, with β-actin as the internal control. **E**, **F** IF staining of the subcellular localization of AKT and p-AKT (Ser473) in LY6H knockdown and overexpression groups compared to their respective control groups in HCCLM3 and Huh7 cells. Red: AKT/p-AKT; Blue: DAPI (nucleus). Scale bar: 50 μm. **G** Western blotting analysis of the rescue effect of IGF-1 (200 ng/mL, 24 h) treatment on LC3B-II and Beclin1 protein expression in LY6H knockdown cells, with β-actin as the internal control. **H** Western blotting analysis of the inhibitory effect of LY294002 (100 μM, 24 h) treatment on LC3B-II and Beclin1 protein expression in LY6H overexpression cells, with β-actin as the internal control. (**I**) IF detection of the effects of IGF-1 treatment on the expression and localization of LC3B and p62 (red). Blue: DAPI (nucleus). Scale bar: 50 μm. **J** TEM analysis of the effects of LY294002 treatment on autophagosome formation (red arrows indicate autophagosomes). Scale bar: 1 μm. Data were displayed as *mean* *±* *SD*. *p < 0.05; **p < 0.01; ***p < 0.001).

Functional validation through qRT-PCR demonstrated that LY6H knockdown reduced expression of PIK3R2 (PI3K), AKT, mTOR, and PRKAA1 (AMPKα1) mRNA (Fig. 3A, S5A). Western blotting analysis showed reduced protein levels of AKT, PI3K, mTOR, and their phosphorylated forms, including p-AKT (Ser473), p-PI3K (Tyr467/Tyr199), and p-mTOR (Ser2448), compared to the control groups (Fig. 3C, S5C). IF staining demonstrated that LY6H knockdown significantly reduced the fluorescence intensity of AKT and p-AKT in HCCLM3 cells and inhibited their nuclear localization (Fig. 3E, S5E). Conversely, LY6H overexpression yielded opposite results in qRT-PCR, Western blotting, and IF assays (Fig. 3B, D, F, S5B, S5D, and S5F).

Pharmacological rescue experiments using IGF-1 (PI3K activator, 200 ng/mL) and LY294002 (PI3K inhibitor, 100 μM) established causality. IGF-1 partially reversed LY6H knockdown-mediated reductions in LC3B-II and Beclin1 (Fig. 3G), while LY294002 blocked LY6H-induced upregulation (Fig. 3H). IF showed IGF-1 increased LC3B puncta and decreased p62 in both control and sh-LY6H cells (Fig. 3I, S5G). TEM confirmed LY294002 reduced autophagosome formation (Fig. 3J, S5H). These data demonstrate LY6H activates autophagy through PI3K/AKT signaling in HCC.

### LY6H binds to PI3K p85 and stabilizes its phosphorylation at Tyr467

**Fig. 4:**
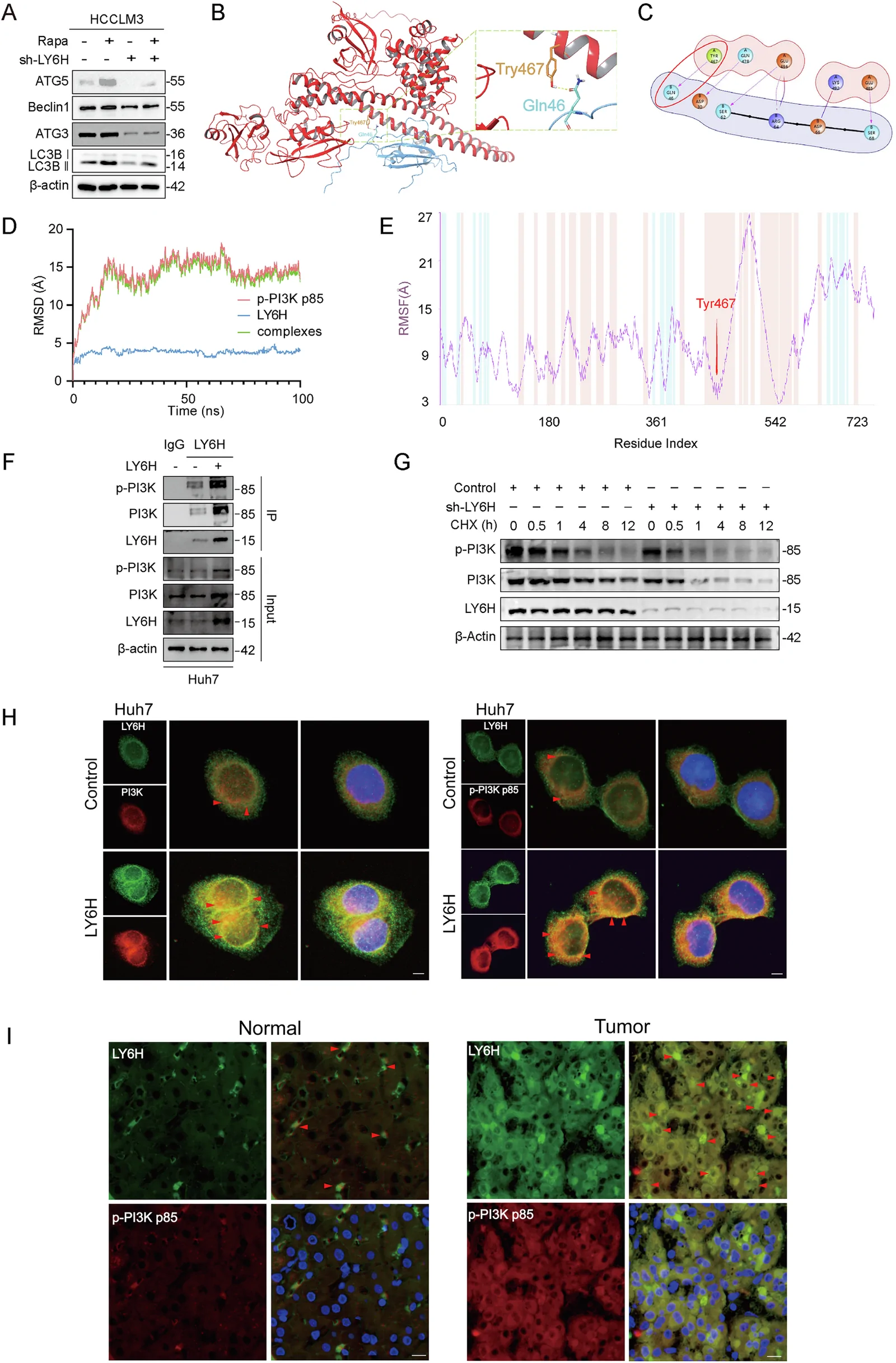
LY6H binds to PI3K p85 and stabilizes its phosphorylation at Tyr467. **A** Western blotting analysis was conducted to assess the protein expression levels of ATG3, ATG5, Beclin1, and LC3B-II in rapamycin-treated HCCLM3 cells, comparing LY6H-knockdown and control groups, with β-actin used as the loading control. **B** Molecular docking model illustrating the binding conformation between LY6H (blue curve) and the p85 subunit of PI3K (red ribbon), with the green region highlighting the Tyr467 phosphorylation site. **C** Two-dimensional interaction diagram depicts hydrogen bonding interactions (red dashed lines) between Gln46 of LY6H and Tyr467 of the p85 subunit of p-PI3K. **D** Root-mean-square-deviation (RMSD) values for the LY6H-PI3K complex during a 100 ns molecular dynamics simulation. **E** Root-mean-square-fluctuation (RMSF) analysis of each residue in the p85 subunit of PI3K, with an arrow indicating the low fluctuation (<1.5 Å) characteristic of the Tyr467 site. **F** Co-immunoprecipitation assay detecting the interaction between LY6H and p-PI3K (Tyr467) in Huh7 cells. LY6H antibody was used for precipitation (with IgG as the control), and Western blotting was employed to detect the expression of p-PI3K (Tyr467) and total PI3K. **G** Determination of the half-life of phosphorylated p85 (p-p85, Tyr467) using the CHX chase assay followed by Western blotting analysis. **H** IF colocalization analysis showing the subcellular distribution of LY6H (green) and PI3K or p-PI3K (red) in Huh7 cells, with DAPI (blue) labeling the nuclei. Scale bar: 50 μm. The red arrow indicates colocalization signals. **I** IF analysis of clinical specimens showed colocalization signals (yellow) of LY6H (green) and p-PI3K (red) in tumor tissues compared to adjacent normal tissues. Nuclei were counterstained with DAPI (blue). Scale bar: 50 μm. Red arrows indicate colocalization signals. Data were displayed as *mean* *±* *SD*.

LY6H promotes autophagy by regulating the PI3K/AKT signaling pathway, with mTORC1, a key downstream effector of this pathway, typically involved in autophagy regulation. However, Western blotting analysis revealed that inhibiting mTORC1 with rapamycin enhanced the expression of autophagy-related molecules (Fig. 4A), suggesting that LY6H may activate autophagy through a mechanism independent of mTOR pathway inhibition.

Based on the observation that LY6H overexpression specifically modulates the phosphorylation of Tyr467 on the PI3K p85 subunit but not Tyr199 on the p55 subunit, we focused on the p85 subunit for downstream experiments (Fig. S5I). Previous studies have shown that the LY6H protein can recognize and bind specific molecules, thereby initiating intracellular signaling events, including protein kinase activation and calcium ion flux, which influence cellular behavior and function [21]. Based on these observations, we hypothesized that LY6H could directly interact with components of the PI3K/AKT pathway. Molecular docking simulations indicated that Gln46 of LY6H directly binds the Tyr467 site of the p85 subunit of PI3K (Fig. 4B, C). This interaction was further supported by molecular dynamics simulations, which demonstrated stable conformational binding between LY6H and PI3K p85 (Fig. 4D, E). Co-immunoprecipitation assays in Huh7 cells revealed that LY6H binds both PI3K and phosphorylated PI3K p85 (Tyr467), and that this interaction was significantly enhanced upon LY6H overexpression (Fig. 4F). Given that LY6H lacks intrinsic kinase activity, we employed cycloheximide (CHX) chase assays to determine the half-life of phosphorylated PI3K (p-PI3K) and to investigate how LY6H regulates its phosphorylation status. CHX (35 μM) chase assays revealed that the protein half-life of phosphorylated p85 (p-p85, Tyr467) was significantly reduced in LY6H-knockdown cells (Fig. 4G). IF analysis revealed that LY6H, PI3K, and its phosphorylated form (p-PI3K) were predominantly localized in the cytoplasm. In Huh7 cells, LY6H overexpression significantly enhanced the colocalization of LY6H with PI3K, as well as with p-PI3K (Fig. 4H). Moreover, IF analysis of clinical tissue specimens demonstrated significantly higher expression levels of both LY6H and p-PI3K in tumor tissues compared with adjacent normal tissues, with pronounced colocalization observed in malignant regions (Fig. 4I). Collectively, these findings suggest that LY6H may stabilize phosphorylated PI3K by binding to p85 (Tyr467), thereby shielding this site from degradation and consequently activating the PI3K/AKT signaling pathway.

### LY6H promotes HCC progression via PI3K/AKT-dependent autophagy

**Fig. 5:**
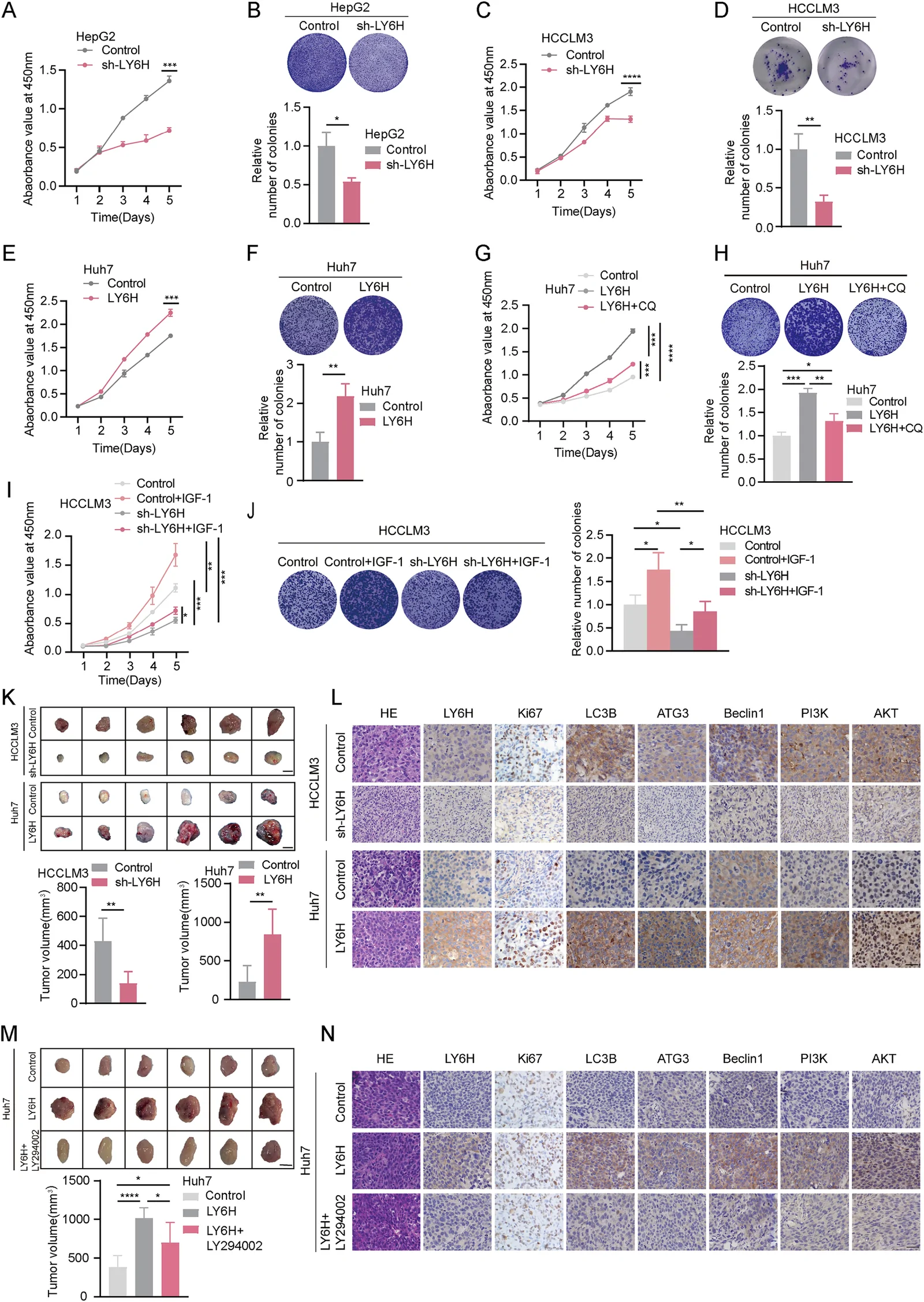
LY6H promotes HCC progression via PI3K/AKT-dependent autophagy. **A** CCK-8 assay results showing cell viability in HepG2 cells with LY6H knockdown versus controls (*n* = 3). **B** Colony formation assays in HepG2 cells with LY6H knockdown versus controls. (Top) Representative images; (Bottom) quantification of colony numbers (*n* = 3). **C** CCK-8 assay results showing cell viability in HCCLM3 cells with LY6H knockdown versus controls (*n* = 3). **D** Colony formation assays in HCCLM3 cells with LY6H knockdown versus controls. (Top) Representative images; (Bottom) quantification of colony numbers (*n* = 3). **E** CCK-8 assay results showing cell viability in Huh7 cells with LY6H overexpression versus controls (*n* = 3). **F** Colony formation assays in Huh7 cells with LY6H overexpression versus controls. (Top) Representative images; (Bottom) quantification of colony numbers (*n* = 3). **G** CCK-8 assay for cell viability in LY6H-overexpressing Huh7 cells treated with CQ (100 μM) (*n* = 3). **H** Colony formation assay in LY6H-overexpressing Huh7 cells treated with CQ (100 μM). (Top) Representative images; (Bottom) quantification analysis of colony numbers (*n* = 3). **I** CCK-8 assay results showing cell viability in LY6H-knockdown HCCLM3 cells with/without PI3K activation versus controls (*n* = 3). **J** Colony formation assays in LY6H-knockdown HCCLM3 cells with/without PI3K activation versus controls. (Left) Representative images; (Right) quantification of colony numbers (*n* = 3). **K** Subcutaneous xenograft tumor experiments in nude mice (n = 6 per group). (Top) Representative images of samples (scale bar: 5 mm); (Bottom) Statistical analysis of tumor volumes. **L** HE and immunohistochemical (IHC) staining of autophagy-related markers and PI3K/AKT pathway proteins in xenograft tumor tissues: HE staining; expression of LY6H, Ki67, LC3B, ATG3, Beclin1, PI3K, and AKT (scale bar: 50 μm). **M** Subcutaneous xenograft tumor experiments in nude mice treated with LY294002 (a PI3K inhibitor) (*n* = 6 per group); (Top) Representative images of samples from treatment groups (scale bar: 5 mm). (Bottom) Statistical analysis of tumor volumes. **N** HE and IHC staining of autophagy-related markers and PI3K/AKT pathway proteins in xenograft tumor tissues after LY294002 treatment: HE staining; expression of LY6H, Ki67, LC3B, ATG3, Beclin1, PI3K, and AKT (scale bar: 50 μm). Data were displayed as *mean* *±* *SD*. (*p < 0.05; **p < 0.01; ***p < 0.001; ****p < 0.0001).

Considering the critical role of LY6H in promoting autophagy in HCC and its association with malignant progression, we further explored the tumor-promoting functions of LY6H and its relationship with autophagy. Clonogenic and Cell Counting Kit-8 (CCK-8) assays demonstrated that LY6H knockdown significantly reduced cell proliferation and colony formation compared to the control group (Fig. 5A–D). Conversely, LY6H overexpression enhanced cell proliferation and colony formation ability, as evidenced by shorter proliferation times and larger colony cluster areas in HCC cells (Fig. 5E, F). To validate the necessity of autophagy in LY6H-driven proliferation, chloroquine (CQ), an autophagy inhibitor, was applied and was found to reverse the increase in cell proliferation and colony formation induced by LY6H overexpression (Fig. 5G, H). Moreover, wound healing and Transwell assays showed that LY6H knockdown impaired cell invasion and migration, whereas overexpression of LY6H resulted in enhanced invasion and migration capabilities (Fig. S6A, B, E, F, I, J). In addition, LY6H-knockdown cells exhibited a higher apoptosis rate, while LY6H-overexpressing cells displayed the opposite result (Fig. S6C, D, G, H, K, L). Crucially, pharmacological activation of PI3K signaling rescued the tumor-suppressive effects of LY6H knockdown (Fig. 5I, J, S6M, and S6N).

We further investigated the role of LY6H in tumorigenesis in vivo by subcutaneously injecting HCC cells into nude mice. Subcutaneous tumor growth was significantly inhibited in HCCLM3 cells transfected with LV-sh-LY6H compared to the control group. In contrast, Huh7 cells transfected with LV-LY6H formed tumors more readily than the control group (Fig. 5K, S7A, and S7B). Immunohistochemical (IHC) staining of autophagy-related markers and signaling pathway proteins in tumor tissues from nude mice revealed that LY6H knockdown inhibited the expression levels of autophagy markers (LC3B, ATG3, and Beclin1), downregulated key molecules in the PI3K/AKT signaling pathway (including PI3K and AKT), and reduced Ki67 proliferation index. Conversely, LY6H overexpression produced the opposite effects (Fig. 5L, S7C, and S7D). Treatment with the PI3K/AKT inhibitor LY294002 significantly suppressed tumor growth after two weeks of in vivo administration (Fig. 5M, S7E). IHC analysis confirmed the suppression of LY6H overexpression–driven pro-tumor effects (Fig. 5N, S7F), and hematoxylin and eosin (H&E) staining revealed no significant organ toxicity (Fig. S7G). Collectively, these findings confirm that the PI3K/AKT pathway plays a critical role in mediating LY6H-driven tumorigenesis.

### NSC243928 suppresses LY6H-driven oncogenic phenotypes in HCC

**Fig. 6:**
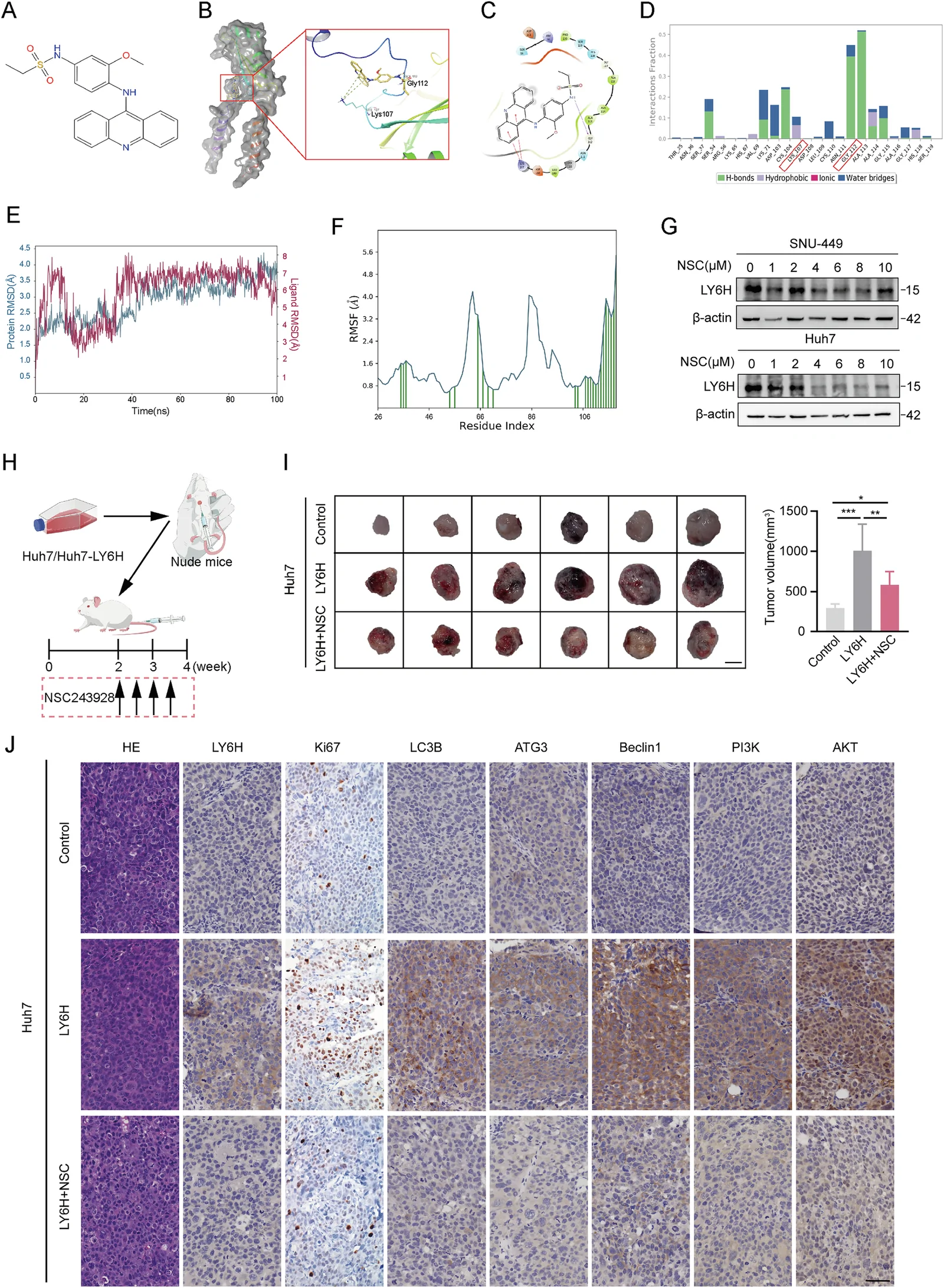
NSC243928 suppresses LY6H-driven oncogenic phenotypes in HCC. **A** Schematic representation of the chemical structure of NSC243928 (molecular weight: 428.5 g/mol). **B** Molecular docking model depicting the binding conformation of NSC243928 to LY6H. **C** Two-dimensional interaction diagram of NSC243928 bound to LY6H. **D** Binding pocket surface depicts non-covalent interactions mediated by NSC243928 at residues Lys107 and Gly112 of LY6H. **E** RMSD values of the NSC243928 (red curve)-LY6H (blue curve) complex during a 100 ns molecular dynamics simulation. **F** Interaction fingerprint map showing persistent contacts between LY6H residues and NSC243928 (green vertical bars indicate contact frequency > 75%). **G** Western blotting analysis of the inhibitory effect of NSC243928 (4 μM, 48 h) on LY6H protein expression, with β-actin used as the internal control. **H** Schematic diagram of the nude mouse subcutaneous xenograft model with subsequent intratumoral NSC243928 administration. **I** Subcutaneous xenograft tumor experiment in nude mice (*n* = 6 per group). (Left) Representative images of samples from treatment groups. (Right) Statistical analysis of tumor volumes (scale bar: 5 mm). **J** HE and IHC staining of xenograft tumor tissues: HE staining; expression of LY6H, Ki67, LC3B, ATG3, Beclin1, PI3K, and AKT (scale bar: 50 μm). Data were displayed as *mean* *±* *SD*. (*p < 0.05; **p < 0.01; ***p < 0.001).

Building on previous evidence that NSC243928, a selective inhibitor of LY6K, induces immunogenic cell death in mammary samples [22–24], we examined its potential cross-reactivity with LY6H in HCC, given the structural homology among LY6 family proteins and the compound’s molecular architecture (Fig. 6A). Molecular docking simulations predicted that NSC243928 stably binds to LY6H through hydrogen bonds and hydrophobic interactions with Gly112 and Lys107 residues of LY6H (Fig. 6B–D). This interaction was further confirmed by molecular dynamics simulations, which showed complex stabilization after 40 ns (Fig. 6E, F). Western blotting analysis demonstrated that 48 h NSC243928 treatment dose-dependently reduced LY6H protein levels compared to controls (Fig. 6G). Functional assays revealed that 4 μM NSC243928 (48 h) effectively suppressed LY6H-driven oncogenic behaviors, including colony formation, invasion (Transwell assay), and migration (wound healing). Flow cytometry analysis further demonstrated that NSC243928 enhanced apoptosis induction (Fig. S8A–F). To evaluate its effects on autophagy, we performed immunofluorescence, transmission electron microscopy, and western blotting analyses, which collectively revealed that NSC243928 inhibits autophagy in HCC cells (Fig. S8G–I). In vivo, administration of NSC243928 for 2 weeks significantly reduced tumor growth, with a 42% decrease in tumor volume in LY6H-overexpressing Huh7 xenografts (Fig. 6H, I, and S8J). IHC analysis of tumor tissues confirmed that NSC243928 downregulated LY6H expression, concomitant with decreased levels of autophagy markers (e.g., ATG3, Beclin1), suppressed PI3K/AKT pathway activation, and reduced Ki67 proliferation index (Fig. 6J, S8K). Notably, HE staining revealed no observable toxicity in major organs (Fig. S9). These findings establish NSC243928 as a potent LY6H inhibitor capable of blocking HCC progression by disrupting the LY6H-PI3K/AKT-autophagy axis, highlighting its therapeutic potential in LY6H-driven HCC.

### LY6H collaborates with autophagy-related proteins and PI3K/AKT pathway components to drive HCC progression and predict poor prognosis

**Fig. 7:**
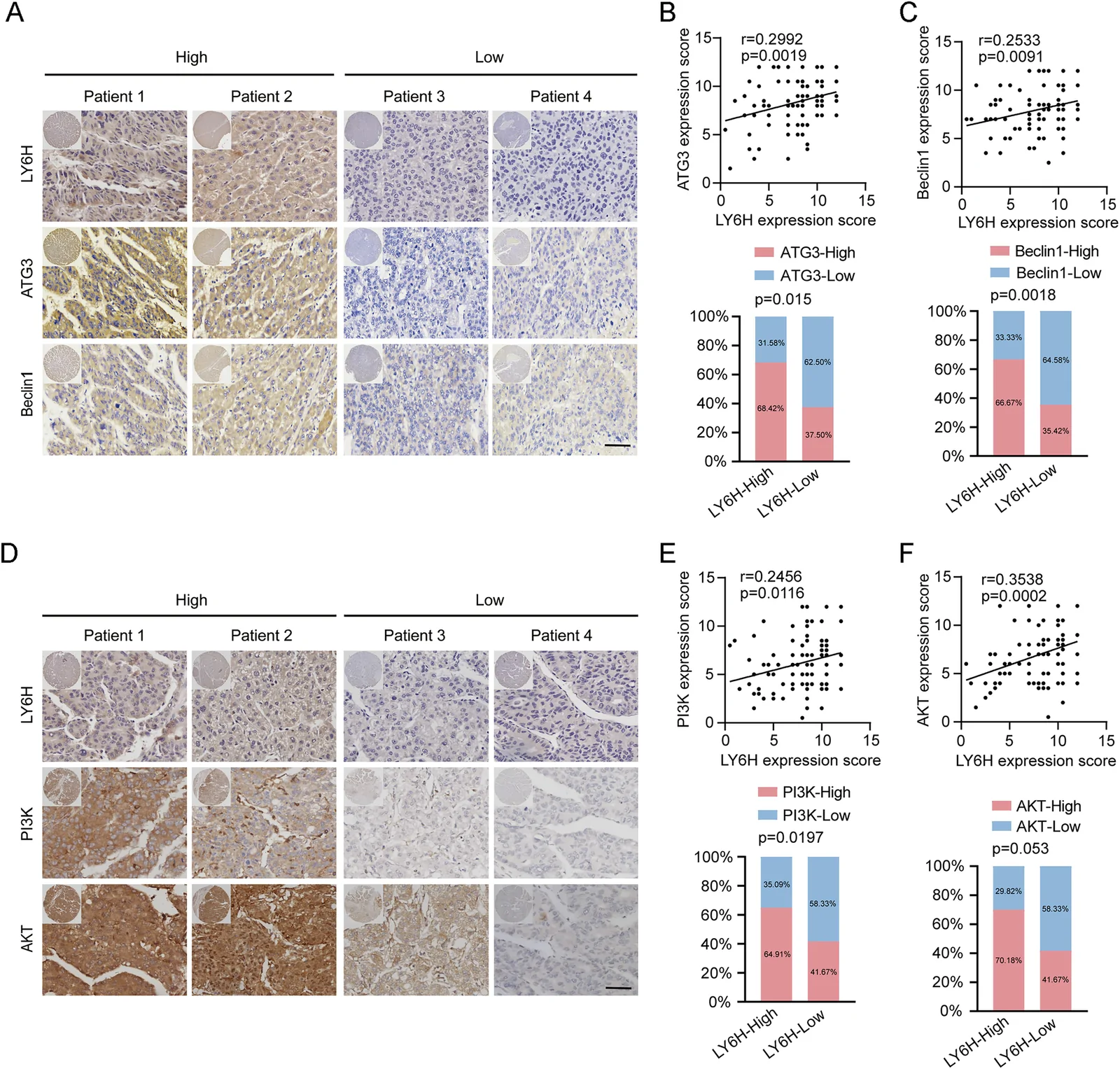
Coordinated expression patterns of LY6H with autophagy-related proteins and PI3K/AKT pathway components in HCC. **A** Representative IHC images displaying co-expression patterns of LY6H and ATG3/Beclin1 in HCC tissues (400× magnification; scale bar = 50 μm). **B** LY6H-ATG3 expression correlation (Pearson’s r and Fisher’s exact test). **C** LY6H-Beclin1 expression correlation (Pearson’s r and Fisher’s exact test). **D** Representative IHC images displaying co-expression patterns of LY6H and PI3K/AKT in HCC tissues (400× magnification; scale bar = 50 μm). **E** LY6H-PI3K correlation analysis (Pearson’s r and Fisher’s exact test). **F** LY6H-AKT correlation analysis (Pearson’s r and Fisher’s exact test). Data were displayed as *mean* *±* *SD*.

IHC analysis of 105 HCC tissue samples assessed LY6H, ATG3, Beclin1, PI3K, and AKT expression. Coordinated expression patterns were observed: LY6H, ATG3, and Beclin1 were frequently co-upregulated or co-downregulated (Fig. 7A); while LY6H, PI3K, and AKT demonstrated co-expression (Fig. 7D). Pearson correlation analysis revealed positive associations between LY6H and ATG3 (*r* = 0.2992); LY6H and Beclin1 (*r* = 0.2533); LY6H and PI3K expression (r = 0.2456); LY6H and AKT expression (*r* = 0.3538). Fisher’s exact test further supported these correlations (Fig. 7B, C, E, F).

Subsequently, we analyzed clinical follow-up records of 105 HCC patients to investigate correlations between LY6H expression and clinicopathological features, including tumor size, HBV infection, cirrhosis, microvascular invasion, and other relevant parameters. The analysis revealed that high LY6H expression significantly correlated with: tumor size (*p* = 0.0301), microvascular invasion (*p* = 0.0217) and Edmondson classification (*p* = 0.0356) (Supplementary Table 2).

**Fig. 8:**
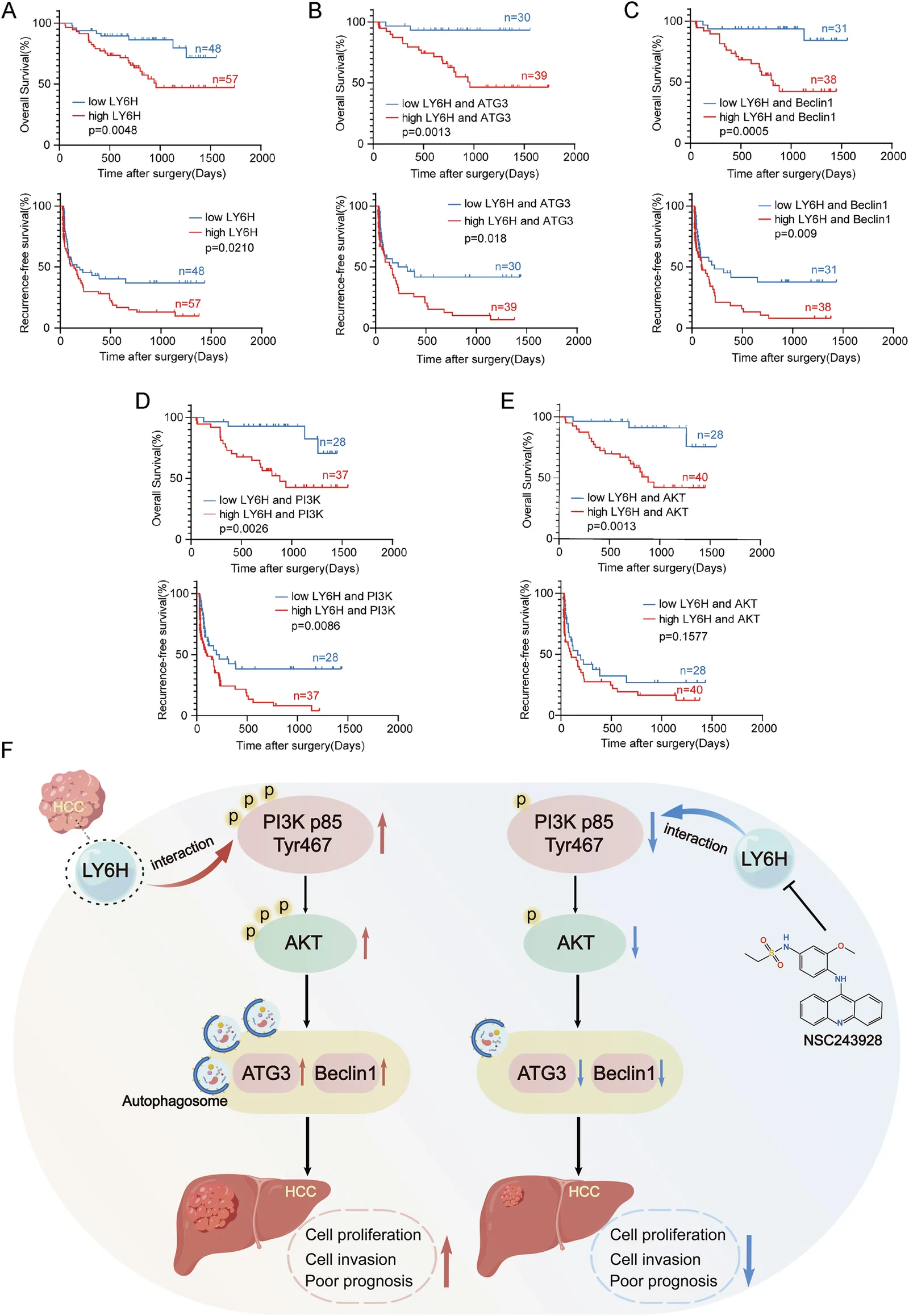
Prognostic value of LY6H and associated molecular signatures in HCC patient outcomes. **A** Kaplan–Meier analysis of LY6H expression groups: Top: Overall survival (OS) of HCC patients (log-rank test). Bottom: Recurrence-free survival (RFS) of HCC patients (log-rank test). **B** OS and RFS of HCC patients based on dual-marker (LY6H/ATG3) expression (log-rank test). **C** OS and RFS of HCC patients based on dual-marker (LY6H/Beclin1) expression (log-rank test). **D** OS and RFS of HCC patients based on dual-marker (LY6H/PI3K) expression (log-rank test). **E** OS and RFS of HCC patients based on dual-marker (LY6H/AKT) expression (log-rank test). **F** LY6H drives autophagy and autophagy-related malignant progression in HCC by binding to PI3K p85 and stabilizing its tyrosine 467 phosphorylation state, while NSC243928 attenuates these effects.

Additionally, using postoperative 3-year follow-up data, Kaplan-Meier survival analysis showed that patients in the high LY6H expression group had significantly shorter 3-year OS and RFS compared to those in the low LY6H expression group (Fig. 8A). Notably, when LY6H was highly co-expressed with ATG3, Beclin1, PI3K, or AKT, patient prognosis further deteriorated, with significantly shorter 3-year OS and RFS compared to the co-low expression groups (Fig. 8B–E). These findings suggest that the synergistic overexpression of LY6H, together with autophagy-related molecules (ATG3, Beclin1) and key components of the PI3K/AKT signaling pathway (PI3K, AKT), may drive HCC progression by modulating tumor cell behavior, thereby providing substantial clinical prognostic value.

## Discussion

LY6H and its family members are markedly overexpressed in a range of malignancies—including pancreatic, ovarian, and colorectal cancers [25–29]—and our group has similarly documented elevated LY6H levels in HCC. Yet its oncogenic mechanisms remained unclear. Guided by second-generation transcriptomic differential enrichment analysis, we centered on autophagy—a critical survival process in the nutrient- and stress-challenged tumor microenvironment [30]—to dissect LY6H’s role in HCC. Clinically, high LY6H expression correlates with poor prognosis, and functionally, we demonstrate that LY6H serves as a pivotal switch driving autophagic flux and thereby promoting HCC progression.

At the mechanistic level, the PI3K/AKT signaling pathway is frequently aberrantly activated in HCC owing to PIK3CA gene amplification or PTEN loss; however, its canonical downstream effector mTORC1 typically suppresses autophagosome formation [31–35]. Notably, although rapamycin-mediated mTORC1 inhibition enhances autophagy, LY6H-dependent autophagy activity persists. This suggests that LY6H may activate autophagy through a mechanism independent of mTOR pathway inhibition, potentially representing a non-canonical regulatory pathway. Here, we demonstrate that LY6H, in a kinase-independent manner, stabilizes phosphorylated PI3K p85 at the Tyr467 site, thereby promoting PI3K/AKT pathway activation. This finding broadens current understanding of the non-canonical cytoplasmic functions of GPI-anchored proteins. Emerging evidence indicates that other GPI-anchored proteins can participate in intracellular signal transduction through processes such as endocytosis. For example, Mizoo et al. reported that, in colorectal cancer, GPLD1 cleaves the GPI anchor of PRSS8, enabling its release from the plasma membrane and subsequent activation of Wnt signaling to induce epithelial–mesenchymal transition [36]. These observations provide conceptual support for the cytoplasmic interaction between LY6H and p85. Previous studies have also established that this site (Tyr467, historically referred to as Tyr458) is critical for LY6H-mediated activation of the PI3K/AKT pathway [37, 38]. While current research has primarily focused on the validated Tyr467 site, systematic exploration of additional interaction interfaces will be an important direction for future investigation. This study found that the LY6H interaction with phospho-PI3K p85 not only amplifies PI3K/AKT signaling through a positive-feedback loop but also bypasses mTORC1-mediated inhibitory regulation, thereby sustaining autophagic activity and consequently promoting HCC progression (Fig. 8F). In summary, this study identifies a previously unrecognized LY6H–p85 PI3K signaling axis that regulates autophagy and malignant progression in HCC, providing a conceptual framework for therapeutic strategies targeting LY6H.

Building on evidence that NSC243928 selectively inhibits LY6K, we evaluated its ability to target LY6H in HCC. Molecular docking confirmed high binding affinity for LY6H, and molecular dynamics simulations demonstrated a stable ligand–receptor complex. Functionally, NSC243928 reduced LY6H protein levels and suppressed HCC cell proliferation, invasion, migration, and autophagic activity. Clinically, patients with concomitant high expression of LY6H and either autophagy markers (e.g., ATG3, Beclin1) or PI3K/AKT pathway components exhibited significantly poorer prognosis than those with low expression (Fig. 8F). These results nominate LY6H and its downstream effectors as prognostic biomarkers and therapeutic targets in HCC, pending clinical validation.

Through comprehensive in vitro and in vivo analyses, we have established the LY6H–PI3K/AKT axis as a key regulator of autophagy in HCC. Nevertheless, several mechanistic questions remain to be addressed, including whether LY6H directly interferes with the interaction between phosphorylated p85 and specific phosphatases or E3 ubiquitin ligases to maintain its phosphorylation state. In addition, although NSC243928 exhibits promising antitumor activity in preclinical models, its pharmacokinetic properties, potential off-target effects, and therapeutic efficacy in combination with existing HCC treatments warrant systematic evaluation in more clinically relevant settings, such as advanced in vivo models or patient-derived xenografts.

In summary, this study provides a novel perspective on LY6H’s role in autophagy and malignant progression of HCC, highlighting the therapeutic potential of disrupting the LY6H-PI3K/AKT axis. Further in-depth investigation is required to address current limitations and advance these findings toward patient application.

## Materials and methods

### Clinical samples and data

Paraffin-embedded tissue samples were collected from 105 patients who underwent hepatocellular carcinoma (HCC) resection at Mengchao Hepatobiliary Hospital of Fujian Medical University between May 2015 and December 2018 (see Supplementary Table 1 for details). The inclusion criteria for tissue sample selection were as follows: (1) Patients underwent radical hepatectomy, with postoperative pathology confirming a diagnosis of HCC; (2) No prior antitumor treatments were administered before surgery. Overall survival (OS) was defined as the time from surgery to patient death, while recurrence-free survival (RFS) was defined as the time from surgery to the first postoperative recurrence. The 3-year OS was calculated from the date of patient death due to HCC or the last follow-up, and the 3-year RFS was calculated from the date of HCC recurrence or the absence of recurrence at the last follow-up. Sample collection, acquisition of clinicopathological data, and patient follow-up were conducted in accordance with the guidelines of the Ethics Committee. Patients were informed of the study’s purpose and procedures prior to the collection of information and samples, and the study was approved by the Fujian Medical Ethics Review Committee under Ethics Approval No. 2025-235.

### IHC staining and result interpretation criteria

Tissue microarrays (TMAs) were constructed using 105 HCC tumor tissues and their paired normal liver tissues. For immunohistochemistry (IHC) staining, tissue sections were dewaxed in xylene and rehydrated through a graded ethanol series. Endogenous peroxidase activity was blocked with 3% hydrogen peroxide, followed by antigen retrieval in Tris-EDTA (pH 9.0) or 10 mM sodium citrate buffer (pH 6.0). After blocking with 5% bovine serum albumin (BSA), sections were incubated with primary antibodies overnight at 4 °C. The sections were then incubated with secondary antibodies for 1 h, visualized using 3,3’-diaminobenzidine (DAB), and counterstained with hematoxylin. Differentiation was performed using hydrochloric acid-alcohol. Sections were mounted with neutral gum and observed under a microscope. IHC-stained sections were scanned using the KF-PRO-005-EX digital slide scanner and analyzed with K-Viewer software. Cell staining was evaluated at 40× magnification. The antibodies used are listed in Supplementary Table 3. Staining intensity was scored on a 4-point scale: 0 (no staining), 1 (light yellow), 2 (brownish yellow), and 3 (brown). The percentage of positively stained cells was scored on a 4-point scale: 0 ( < 5%), 1 (5%–25%), 2 (26%–50%), 3 (51%–75%), and 4 (76%–100%). The final score was calculated as the product of the intensity and percentage scores.

### Real-time quantitative polymerase chain reaction (qRT-PCR)

Total RNA extracted from HCC cells was reverse transcribed using the Prime Script RT reagent kit (Takara Bio, Tokyo, Japan). qRT-PCR was performed using TB Green Premix Ex Taq (Takara Bio, Tokyo, Japan) on a CFX Real-Time PCR Detection System (Bio-Rad, Hercules, CA, USA), following the manufacturer’s instructions. Relative mRNA levels were calculated using the 2⁻ΔΔCt method, with the 18S gene serving as the internal reference. (The primers used in this experiment are listed in Supplementary Table 4.)

### Western blotting analysis

Protein extraction was performed using RIPA lysis buffer supplemented with protease inhibitors (Beyotime Biotechnology, Shanghai, China). Protein samples (20–30 μg) were separated by electrophoresis on 10% or 12% SDS-polyacrylamide gels and transferred to PVDF membranes (Millipore, USA). Membranes were blocked with 5% non-fat milk in TBST for 1.5 h at room temperature, followed by overnight incubation at 4 °C with primary antibodies (see Supplementary Table 3 for details). After washing, membranes were incubated with HRP-conjugated secondary antibodies (Boster Biological Technology/Invitrogen) for 1.5 h at room temperature. β-actin (Cell Signaling Technology; USA) served as the loading control. Protein bands were visualized using Enhanced BeyoECL Star Detection Reagent (Beyotime Biotechnology, Shanghai, China), and densitometric analysis was performed using ImageJ software (NIH).

### Immunofluorescence (IF) staining

Cells were seeded in 24-well plates at a density of 2 × 10⁴ cells per well and cultured until they reached the appropriate confluence. Cells were fixed with 4% paraformaldehyde at room temperature for 30 min and permeabilized with 0.2% Triton X-100 for 15 min. After blocking with 5% fetal bovine serum for 1 h, primary antibodies were added and incubated overnight at 4 °C. Following three washes with phosphate-buffered saline containing 0.1% Tween-20 (PBST), fluorescent secondary antibodies (goat anti-rabbit IgG-Alexa Fluor 594 or goat anti-mouse IgG-Alexa Fluor 488, CST, USA) were incubated at room temperature for 1 h. Cell nuclei were stained with 4’,6-diamidino-2-phenylindole dihydrochloride (DAPI). Images were acquired using a confocal microscope (Olympus FV1000, Japan). The antibodies used are listed in Supplementary Table 3.

### Cell preparation for transmission electron microscopy (TEM)

Cultured cells were harvested by digestion and centrifugation at 1000 rpm for 5 min. The resulting cell pellets were fixed in a solution of 3% glutaraldehyde and 1.5% paraformaldehyde at 4 °C for several days. The fixation solution was then replaced with 1% osmium tetroxide and 1.5% potassium ferricyanide for 1.5 h, followed by multiple washes with phosphate-buffered saline (PBS). Subsequently, the sections were stained with uranyl acetate saturated in 70% ethanol, dehydrated using an ethanol-acetone gradient, and embedded in Epoxy Resin 618. Ultrathin sections (90 nm) were stained with uranyl acetate and lead citrate for 5–15 min each, and were then examined and imaged using an FEI TECNAI transmission electron microscope.

### Molecular docking and molecular dynamics simulation

Molecular docking simulations were performed using Schrödinger software. The protein structure of the target protein LY6H (UniProt accession: O94772) and PI3K p85 (UniProt accession: P27986) was obtained from the UniProt database (https://www.uniprot.org) and optimized. The receptor grid was generated using the software, and the structure of NSC243928 was drawn and optimized using LigPrep before docking. Ligand docking was performed using Glide in standard precision (SP Docking) mode, with other parameters set to default values. The resulting compound-protein complexes from the docking were analyzed using Maestro.

Molecular dynamics (MD) simulations were conducted with the Desmond module (v53011, Schrödinger). The complexes were prepared using the Protein Preparation module, minimized, and optimized before being imported into the System Builder module. Solvation was performed using the TIP3P water model and OPLS3 force field, with Na+ ions added for neutralization. After energy minimization and system equilibrium, MD simulations were run for over 100 ns. Finally, the simulation output files were analyzed using the Simulation Interactions Diagram function. Root mean square deviation (RMSD) was used to quantify the average displacement of selected atoms relative to a reference frame.

### Immunoprecipitation

HCC cells were lysed with RIPA buffer (containing protease and phosphatase inhibitors) at 4 °C for 30 min. 2 μg of anti-LY6H antibody or IgG control was added, and the mixture was incubated overnight at 4 °C with gentle rotation. Fifty microliters of Protein A/G magnetic beads were then added and incubated at 4 °C for 2 h. The beads were washed four times with pre-chilled PBS (1000 g, 4 °C, 5 min). SDS loading buffer (2×) was then added, and the samples were boiled at 95 °C for 10 min. Western blotting analysis was performed using anti-PI3K or anti-p-PI3K antibody. (The antibodies used are listed in Supplementary Table 3.)

### Animal experiments

Three- to five-week-old male BALB/c nude mice (purchased from Shanghai SLAC Laboratory Animal Co., Ltd) were used as the animal model. The mice were housed in an SPF-grade barrier facility at Fujian Medical University. Experimental groups were randomly assigned, with a minimum of six animals per group. In the subcutaneous tumor model, 100 μL of HCC cell suspension (2 × 10⁶ cells) was injected subcutaneously into the axilla of the nude mice. Tumor length (L) and width (W) were measured every 4 days, and tumor volume was calculated using the formula: Volume (mm³) = (W² × L)/2. After 4 weeks, the mice were sacrificed, and tumor tissues were excised, fixed, and embedded in paraffin for further analysis. All animal procedures were conducted in compliance with the NIH Guidelines for the Care and Use of Laboratory Animals and were approved by the institutional ethics committee (Ethics No.: IACUC FJMU 2024-0417).

Two weeks after subcutaneous injection of Huh7 cells and LV-LY6H-infected Huh7 cells, the subcutaneous tumor models were successfully established. The mice were then treated with the LY6H inhibitor NSC243928 (30 mg/kg) (HY-123885, MCE, USA) via tail vein injection, or with LY294002 (100 mg/kg, twice weekly) (HY-10108, MCE, USA) via intraperitoneal injection. After 4 weeks, the mice were euthanized, and their livers and other vital organs were excised. Tumor volume and the presence of metastases were recorded. Tumor volume was calculated using the formula: Volume (mm³) = (W² × L)/2, where L and W represent the tumor’s long and short diameters, respectively. Tumor tissues and vital organs were collected for HE staining and IHC.

### Statistical analysis

Statistical analyses were performed using GraphPad Prism 9.0 software. Data are presented as mean ± standard deviation (SD). For normally distributed data with homogeneous variances, one-way analysis of variance (ANOVA) was used to compare means across multiple groups, followed by the LSD post-hoc test for pairwise comparisons. Associations between categorical variables were assessed using Pearson’s chi-square test and Fisher’s exact test. Survival curves were generated using the Kaplan-Meier method, and differences in survival times were analyzed using the log-rank test. Statistical significance was defined as a two-sided *p* < 0.05. Additional details on materials and methods can be found in the Supplementary information.

## Supplementary information

Supplementary Table 5

Supplementary information

Original Western blots

## Data Availability

Data and materials are available by contacting the corresponding author for reasonable requests.
