# Supplementary material for: Targeting the LY6H-PI3K/AKT autophagy axis suppresses HCC malignancy and reveals a druggable vulnerability
Source: Cell Death Dis. 2026 Jun 15;17(1):748. doi: 10.1038/s41419-026-08947-z (PMC13493903; doi:10.1038/s41419-026-08947-z)
Supplement: Supplementary file 1 [file 41419_2026_8947_MOESM2_ESM.docx]

**Supplementary information for**

**Targeting the LY6H-PI3K/AKT Autophagy Axis Suppresses HCC Malignancy and Reveals a Druggable Vulnerability**

Fei Wei **^1,2,#^**, Jie Wang **^1,2,#^**, Ye Cheng **^1,2,#^**, Caiyan Chen **^1,2^**, Yong Wu **^1,^**^2^, Jie Pan **^1,^**^2^, Jiahong Wang **^1,2^**, Yufei Wang **^1,2^**, Meifeng Wang **^1,2^**, Xueni Zeng **^1,^**^2^, Aimin Huang **^1,2^**^*^

**This file includes:**

Supplementary Materials and Methods

Supplementary Figure S1 to S9

Supplementary Table 1 to 4

**Supplementary Materials and Methods**

**Cell Culture and Lentiviral Transfection**

Based on our previous research on LY6H expression in HCC cells, we selected two cell lines with high LY6H expression (HepG2 and HCCLM3) and two with low LY6H expression (SNU-449 and Huh7) for subsequent experiments. All HCC cell lines were provided by the Oncology Laboratory of Fujian Medical University. HepG2 cells were cultured in MEM medium, HCCLM3 and Huh7 cells in high-glucose DMEM medium, and SNU-449 cells in RPMI-1640 medium. All media were supplemented with 10% fetal bovine serum, and cells were passaged using 0.25% EDTA-containing trypsin. Lentiviral packaging was performed to generate cell lines with stable LY6H overexpression or knockdown. At 50% confluency, 293T cells were co-transfected with the appropriate plasmids and lentiviral packaging plasmids REV, RRE, and VSVG (Invitrogen, Carlsbad, CA, USA). Culture medium was collected at 48 and 72 h post-transfection, filtered through a 0.45 μm filter, and added to HCC cells seeded in 6-well plates (1 × 10⁵ cells per well). After 48 hours, cells were selected for 7–14 days using 1 μg/mL puromycin (Beyotime Biotech, Shanghai, China) or 300 μg/mL G418 (Sigma-Aldrich, St. Louis, MO, USA). Cells with stable LY6H overexpression or knockdown were used for subsequent analyses.

**RNA Sequencing and Analysis**

Cell samples of HepG2 sh-LY6H, HCCLM3 sh-LY6H, and their corresponding controls were collected (with three biological replicates), and total RNA was extracted using TRIzol reagent (Invitrogen, Carlsbad, CA, USA). After quality control, sequencing libraries were constructed using a strand-specific mRNA library preparation kit (Wuhan Seqhealth Co., Ltd., China). Sequencing was performed on the Illumina HiSeq platform by Wuhan Seqhealth Co., Ltd. The raw data were processed using the edgeR software package (version 3.12.1) to identify differentially expressed genes (DEGs) between the two groups. The screening criteria included an absolute log₂ fold change > 1 and a p-value < 0.05. Downstream analyses encompassed Gene Ontology (GO) enrichment analysis, Kyoto Encyclopedia of Genes and Genomes (KEGG) pathway analysis, and other bioinformatics approaches.

**Cell Proliferation Assay and Colony Formation Assay**

HCC cells were seeded into 96-well plates at a density of 1 × 10³ cells per well, and cell proliferation was assessed using the CCK-8 assay (HERUI, Fuzhou, China) according to the manufacturer's instructions. After adding the CCK-8 solution, cells were incubated for 1 hour. The absorbance at 450 nm was measured using a microplate reader. Measurements were taken daily for 5 days, and cell growth curves were plotted. For the colony formation assay, 1 × 10³ HCC cells were cultured in 6-well plates for two weeks, until colonies containing ≥50 cells became visible to the naked eye. The colonies were then fixed with paraformaldehyde for 30 minutes, stained with crystal violet (Beyotime Biotech, Shanghai, China) for 1 hour, and subsequently counted.

**Wound Healing Assay**

Horizontal lines were drawn on the back of a 6-well plate at intervals of 0.5-1 cm using a marker pen. Liver cancer cells in the logarithmic growth phase were collected, their concentration adjusted, and seeded into the 6-well plate. The cells were incubated at 37°C with 5% CO₂ for 24 h. Once the cell density reached approximately 80%, a scratch was made perpendicular to the horizontal lines using a pipette tip. The cells were washed twice with phosphate-buffered saline (PBS) to remove detached cells, and serum-free medium was added. The plate was returned to the incubator, and images were captured at 0, 48, and 72 h.

**Transwell cell migration and invasion assay**

HCC cells in the logarithmic growth phase were digested, centrifuged, washed twice with PBS, and resuspended in serum-free medium to a concentration of 1 × 10^5^ cells/300 μL. For migration assays, 300 µL cell suspension was loaded into the upper chamber containing serum-free medium, and complete medium was added to the lower chamber. For invasion assays, Invasion chambers, pre-coated with Matrigel, were equilibrated at room temperature and then incubated with serum-free medium at 37°C for 2 h. After excess medium was carefully aspirated (without disturbing the gel layer), 500 μL cell suspension was added to the upper chamber, and complete medium was placed in the lower chamber. Chambers were collected at 48 h post-seeding. After washing with PBS, cells were fixed with pre-chilled methanol for 15 minutes, followed by an additional PBS wash. Cells were stained with 0.1% crystal violet for 20 minutes and subsequently washed three times with PBS. Non-migrated cells on the upper surface of the chambers were removed using a cotton swab. Chambers were then photographed under an inverted microscope. Each sample was assayed in triplicate, with five random fields per chamber selected for cell counting. A two-tailed t-test was used to compare migration and invasion abilities between the two groups.

**Flow cytometry**

Apoptosis was analyzed using Annexin V-APC/PI Apoptosis Detection Kit (Procell, P-CA-207, Wuhan, China) following the manufacturer's protocol. Briefly: 5 × 10^5^ HCC cells were centrifuged at 300 × g for 5 min, washed twice with PBS, then resuspended in binding buffer containing 5 µL Annexin V-APC and 5 µL PI staining solutions. After 20-min incubation at room temperature under dark conditions, samples were immediately analyzed on Thermo Fisher flow cytometer. Data quantification was conducted using FlowJo software (Treestar, USA). Total apoptotic cells were defined as the sum of early apoptotic (Annexin V^+^, PI^-^) and late apoptotic (Annexin V^+^, PI^+^) cell populations.

**Supplementary Figure S1 to S9**

**Supplementary Figures and Legends**

**Figure S1. Construction of HCC Cell lines with stable LY6H knockdown/overexpression**

(**A**) qRT-PCR was performed to measure the basal mRNA expression levels of LY6H in HCC cell lines, with 18S used as the internal control. (**B**) Western blotting analysis was conducted to assess the basal protein expression levels of LY6H in HCC cell lines, with β-actin serving as the loading control. (**C**) qRT-PCR analysis of LY6H mRNA expression levels in LV-shLY6H-infected HepG2 and HCCLM3 cells, using 18S as the internal control. (**D**) Western blotting analysis of LY6H protein expression levels in LV-shLY6H-infected HepG2 and HCCLM3 cells, with β-actin as the internal control. (**E**) qRT-PCR analysis of LY6H mRNA expression levels in LV-LY6H-infected SNU-449 and Huh7 cells, with 18S as the internal control. (**F**) Western blotting analysis of LY6H protein expression levels in LV-LY6H-infected SNU-449 and Huh7 cells, with β-actin as the internal control. Data were displayed as *mean ± SD*. (*, *p* < 0.05; **, *p* < 0.01; ***, *p* < 0.001; ns, no statistical significance)

**Figure S2. RNA-Seq analysis of HCC cells with LY6H knockdown and control groups**

(**A**) Volcano plot of DEGs in HepG2 cells with LY6H knockdown compared to control cells (|log₂FC| ≥ 1, *p* < 0.05). (**B**) Volcano plot of DEGs in HCCLM3 cells with LY6H knockdown compared to control cells (|log₂FC| ≥ 1, *p* < 0.05). (**C**) GO functional enrichment analysis of DEGs in HepG2 cells with LY6H knockdown compared to control cells.

**Figure S3. Regulation of tumor cell autophagy in HCC by LY6H knockdown/overexpression**

(**A**, **B**) qRT-PCR analysis of ATG3, ATG5, Beclin1, and LC3B expression levels in LY6H knockdown groups versus control groups in HepG2 cells, and in LY6H overexpression groups versus control groups in SNU-449 cells, with 18S as the internal control. (**C**, **D**) Western blotting analysis of ATG3, ATG5, ATG7, Beclin1, and LC3B-II expression levels in LY6H knockdown groups versus control groups in HepG2 cells, and in LY6H overexpression groups versus control groups in SNU-449 cells, with β-actin as the internal control. (**E**, **F**) Quantification of LC3B and p62 fluorescence intensity in HCCLM3 and Huh7 cells under LY6H knockdown/overexpression conditions (n = 3). (**G**, **H**) Quantitative analysis of autophagosome numbers in HCCLM3 and Huh7 cells with LY6H knockdown or overexpression via TEM (n = 3). Data were displayed as *mean ± SD*. (*, *p* < 0.05; **, *p* < 0.01; ***, *p* < 0.001; ****, *p* < 0.0001)

**Figure S4. Association of LY6H with the PI3K/AKT signaling pathway**

(**A**) Results of KEGG pathway enrichment analysis comparing HepG2 cells with LY6H knockdown to control cells, showing significant enrichment of the PI3K/AKT signaling pathway (p < 0.05). (**B**) KEGG pathway enrichment analysis comparing HCCLM3 cells with LY6H knockdown to control cells, also demonstrating significant enrichment of the PI3K/AKT signaling pathway (p < 0.05). (**C**) Pearson correlation analysis of LY6H expression and core genes of the PI3K/AKT pathway (PIK3R6, PIK3CD, AKT1S1, and LAMTOR1, etc.) in the TCGA database.

**Figure S5. Effects of LY6H knockdown/overexpression on the PI3K/AKT signaling pathway**

(**A**) qRT-PCR analysis of PI3K, AKT, AMPK and mTOR mRNA expression levels in LY6H knockdown groups compared to control groups in HepG2 cells, with 18S as the internal control. (**B**) qRT-PCR analysis of PI3K, AKT, AMPK and mTOR mRNA expression levels in LY6H overexpression groups compared to control groups in SNU-449 cells, with 18S as the internal control. (**C**) Western blotting analysis of AKT, PI3K, mTOR, and their phosphorylated forms in LY6H knockdown groups compared to control groups in HepG2 cells, with β-actin as the internal control. (**D**) Western blotting analysis of AKT, PI3K, mTOR, and their phosphorylated forms in LY6H overexpression groups compared to control groups in SNU-449 cells, with β-actin as the internal control. (**E**, **F**) Quantitative analysis of AKT and p-AKT fluorescence intensity in HCCLM3 and Huh7 cells with LY6H knockdown/overexpression (n = 3). (**G**) Quantitative IF analysis of LC3B and p62 expression following IGF-1 treatment (n = 3). (**H**) Quantitative TEM analysis of autophagosome numbers following LY294002 treatment (n = 3). (**I**) In Huh7 cells, Western blotting was used to analyze the expression levels of PI3K subunit p55 in the LY6H overexpression group and the control group, with β-actin as the internal reference. Data were displayed as *mean ± SD*. (*, *p* < 0.05; **, *p* < 0.01; ***, *p* < 0.001)

**Figure S6. LY6H regulates the migration, invasion, and apoptosis of HCC cells via PI3K/AKT pathway**

(**A**) Wound healing assays in HepG2 cells with LY6H knockdown versus controls. (Top) Representative images (0/48 h); (Bottom) quantification of migrated areas (n = 3). (**B**) Transwell migration and invasion assays in HepG2 cells with LY6H knockdown versus controls. (Top) Migration and invasion cell staining; (Bottom) quantification of penetrated cells areas (n = 3). (**C**) Flow cytometry analysis of apoptosis in HepG2 cells with LY6H knockdown versus controls. (Top) Representative scatter plots; (Bottom) quantification of apoptosis rates (n = 3). (**D**) Western blotting analysis of apoptosis in HepG2 cells with LY6H knockdown versus controls. (**E**) Wound healing assays in HCCLM3 cells with LY6H knockdown versus controls. (Top) Representative images (0/48 h); (Bottom) quantification of migrated areas (n = 3). (**F**) Transwell migration and invasion assays in HCCLM3 cells with LY6H knockdown versus controls. (Top) Migration and invasion cell staining; (Bottom) quantification of penetrated cells areas (n = 3). (**G**) Flow cytometry analysis of apoptosis in HCCLM3 cells with LY6H knockdown versus controls. (Top) Representative scatter plots; (Bottom) quantification of apoptosis rates (n = 3). (**H**) Western blotting analysis of apoptosis in HCCLM3 cells with LY6H knockdown versus controls. (**I**) Wound healing assays in Huh7 cells with LY6H overexpression versus controls. (Top) Representative images (0/48 h); (Bottom) quantification of migrated areas (n = 3). (**J**) Transwell migration and invasion assays in Huh7 cells with LY6H overexpression versus controls. (Top) Migration and invasion cell staining; (Bottom) quantification of penetrated cells areas (n = 3). (**K**) Flow cytometry analysis of apoptosis in Huh7 cells with LY6H overexpression versus controls. (Top) Representative scatter plots; (Bottom) quantification of apoptosis rates (n = 3). (**L**) Western blotting analysis of apoptosis in Huh7 cells with LY6H overexpression versus controls. (**M**) Wound healing assays in LY6H-knockdown HCCLM3 cells with/without PI3K activation versus controls. (Top) Representative images (0/72 h); (Bottom) quantification of migrated areas (n = 3). (**N**) Transwell invasion assays in LY6H-knockdown HCCLM3 cells with/without PI3K activation versus controls. (Top) Staining of Matrigel-penetrated cells; (Bottom) quantification of penetrated cells areas (n = 3). Data were displayed as *mean ± SD*. (*, *p* < 0.05; **, *p* < 0.01; ***, *p* < 0.001)

**Figure S7. LY6H drives in vivo tumorigenesis through the PI3K/AKT signaling pathway**

(**A**, **B**) Tumor volume growth curves of subcutaneous xenograft tumors in nude mice (n = 6). (**C**, **D**) Quantitative analysis of protein expression by IHC in subcutaneous xenograft tumors (n = 6). (**E**) Growth curve of subcutaneous tumors in nude mice after LY294002 treatment (a PI3K inhibitor) (n = 6). (**F**) Quantitative analysis of IHC in subcutaneous tumors of nude mice after LY294002 treatment (n = 6). (**G**) HE staining of major organs (heart, liver, spleen, lung, kidney) from tumor-bearing mice treated with LY294002, showing no significant pathological alterations. Scale bar: 50 μm. Data were displayed as *mean ± SD*. (*, *p* < 0.05; **, *p* < 0.01; ***, *p* < 0.001; ****, *p* < 0.0001; ns, no statistical significance)

**Figure S8. NSC243928 inhibits malignant phenotypes of HCC cells**

(**A**) Colony formation assays in LY6H-overexpressing Huh7 cells with/without NSC243928. (Left) Representative images; (Right) quantification of colony numbers (n = 3). (**B**) Flow cytometry analysis of apoptosis in LY6H-overexpressing Huh7 cells with/without NSC243928. (Left) Representative scatter plots; (Right) quantification of apoptosis rates (n = 3). (**C**) Wound healing assays in LY6H-overexpressing Huh7 cells with/without NSC243928. (Left) Representative images (0/48 h); (Right) quantification of migrated areas (n = 3). (**D**) Transwell invasion assays in LY6H-overexpressing Huh7 cells with/without NSC243928. (Left) Staining of Matrigel-penetrated cells; (Right) quantification of penetrated cells (n = 3). (**E**) Wound healing assays in LY6H-overexpressing SNU-449 cells with/without NSC243928. (Left) Representative images (0/48 h); (Right) quantification of migrated areas (n = 3). (**F**) Transwell invasion assays in LY6H-overexpressing SNU-449 cells with/without NSC243928. (Left) Staining of Matrigel-penetrated cells; (Right) quantification of penetrated cells (n = 3). (**G**) IF analysis of LC3B and p62 expression following NSC243928 treatment. (Left) Representative images; (Right) quantification of LC3B puncta and p62 intensity (n = 3). (**H**) TEM analysis of autophagosome numbers following NSC243928 treatment. (Left) Representative images; (Right) quantification of autophagosome density (n = 3). (**I**) Western blotting analysis of autophagy-related protein expression following NSC243928 treatment. (**J**) Growth curves of subcutaneous xenograft tumors in nude mice after NSC243928 treatment (n = 6). (**K**) Quantitative IHC analysis of subcutaneous xenograft tumors in nude mice after NSC243928 treatment (n = 6). Data were displayed as *mean ± SD*. (*, *p* < 0.05; **, *p* < 0.01; ***, *p* < 0.001; ****, *p* < 0.0001; ns, no statistical significance)

**Figure S9. In vivo safety assessment of NSC243928**

HE staining of major organs (heart, liver, spleen, lung, kidney) from tumor-bearing mice treated with NSC243928, showing no significant pathological alterations. Scale bar: 50 μm.

**Supplementary Table 1**

**List of patient clinical information**

| Groups | n | Positive (%) |
| --- | --- | --- |
|  |  |  |
|  |  |  |
| Sex |  |  |
| Male | 91 | 86.67 |
| Female | 14 | 13.33 |
| Age (years) |  |  |
| ≤60 | 67 | 63.81 |
| >60 | 38 | 36.19 |
| Tumor size (cm) |  |  |
| <10 | 57 | 54.29 |
| ≥10 | 48 | 45.71 |
| HBsAg |  |  |
| No | 39 | 37.14 |
| Yes | 66 | 62.86 |
| Cirrhosis |  |  |
| No | 46 | 43.81 |
| Yes | 59 | 56.19 |
| Microvascular invasion |  |  |
| No | 24 | 22.86 |
| Yes | 81 | 77.14 |
| Microsatellites |  |  |
| No | 50 | 47.62 |
| Yes | 55 | 52.38 |
| Edmondson classification |  |  |
| Ⅰ+Ⅱ | 34 | 32.38 |
| Ⅲ+Ⅳ | 71 | 67.62 |
| Total | 105 | 100 |

**Supplementary Table 2**

**List of Clinical relevance of LY6H in HCC**

| Groups | LY6H expression | | n | Positive (%) | p value (Fisher's exact test) |
| --- | --- | --- | --- | --- | --- |
|  |  |  |  |  |  |
|  | Low | High |  |  |  |
| Sex |  |  |  |  |  |
| Male | 41 | 50 | 91 | 86.67 | 0.779 |
| Female | 7 | 7 | 14 | 13.33 |  |
| Age (years) |  |  |  |  |  |
| ≤60 | 32 | 35 | 67 | 63.81 | 0.6843 |
| >60 | 16 | 22 | 38 | 36.19 |  |
| Tumor size (cm) |  |  |  |  |  |
| <10 | 32 | 25 | 57 | 54.29 | 0.0301* |
| ≥10 | 16 | 32 | 48 | 45.71 |  |
| HBsAg |  |  |  |  |  |
| No | 20 | 19 | 39 | 37.14 | 0.4215 |
| Yes | 28 | 38 | 66 | 62.86 |  |
| Cirrhosis |  |  |  |  |  |
| No | 19 | 27 | 46 | 43.81 | 0.4379 |
| Yes | 29 | 30 | 59 | 56.19 |  |
| Microvascular invasion |  |  |  |  |  |
| No | 16 | 8 | 24 | 22.86 | 0.0217* |
| Yes | 32 | 49 | 81 | 77.14 |  |
| Microsatellites |  |  |  |  |  |
| No | 28 | 22 | 50 | 47.62 | 0.0516 |
| Yes | 20 | 35 | 55 | 52.38 |  |
| Edmondson classification |  |  |  |  |  |
| Ⅰ+Ⅱ | 21 | 13 | 34 | 32.38 | 0.0356* |
| Ⅲ+Ⅳ | 27 | 44 | 71 | 67.62 |  |
| Total | 48 | 57 | 105 | 100.00 |  |

Statistically significant; **, p*< 0.05.

**Supplementary Table 3**

**List of primary antibodies for**

**Western blotting, immunohistochemistry, immunofluorescence, and immunoprecipitation**

| **Antibody** | **Catalog Number** | **Application** | **Dilution/Working concentration** | **Company** |
| --- | --- | --- | --- | --- |
| Anti-LY6H antibody | sc-100285 | Western Blotting | 1:1000 | Santa |
|  |  | IHC | 1:200 |  |
|  |  | IF | 1:200 |  |
|  |  | IP | 2μg |  |
| Anti-β-catenin antibody | #4970 | Western Blotting | 1:1000 | CST |
| Anti-PI3K antibody | T40115 | Western Blotting | 1:1000 | Abmart |
|  |  | IHC | 1:200 |  |
|  |  | IF | 1:100 |  |
| Anti-p-PI3K antibody | T40116 | Western Blotting | 1:1000 | Abmart |
|  |  | IF | 1:100 |  |
| Anti-AKT antibody | T55561 | Western Blotting | 1:1000 | Abmart |
|  |  | IHC | 1:200 |  |
|  |  | IF | 1:100 |  |
| Anti-p-AKT antibody | T40067 | Western Blotting | 1:1000 | Abmart |
|  |  | IF | 1:100 |  |
| Anti-mTOR antibody | T55306 | Western Blotting | 1:1000 | Abmart |
| Anti-p-mTOR antibody | T56571 | Western Blotting | 1:1000 | Abmart |
| Atg3 Antibody | #3415 | IHC | 1:1000 | CST |
| Anti-ATG3 antibody | bs-4013R | Western Blotting | 1:1000 | Bioss |
| Atg5 (D5F5U) Rabbit mAb | #12994 | Western Blotting | 1:1000 | CST |
| Atg7 (D12B11) Rabbit mAb | #8558 | Western Blotting | 1:1000 | CST |
| Beclin1 antibody | T55092 | Western Blotting | 1:1000 | Abmart |
|  |  | IHC | 1:100 |  |

**Supplementary Table 3(continued)**

| **Antibody** | **Catalog Number** | **Application** | **Dilution/Working concentration** | **Company** |
| --- | --- | --- | --- | --- |
| LC3B antibody | T55992 | Western Blotting | 1:1000 | Abmart |
|  |  | IHC | 1:100 |  |
|  |  | IF | 1:100 |  |
| p62/SQSTM1 Antibody | T55546 | Western Blotting | 1:1000 | Abmart |
|  |  | IF | 1:100 |  |
| Ki67 | RMA-0731 | IHC | Ready-to-use antibodies for IHC | MXB |
| HRP Conjugated AffiniPure Goat AntiRabbit IgG (H+L) | BA1054 | Western Blotting | 1:7000 | Boster |
| Goat anti-Mouse IgG (H+L) Secondary Antibody; HRP | #31430 | Western Blotting | 1:5000 | Invitrogen |
| Goat Anti-Rabbit IgG H&L (Alexa Fluor® 594) | ab150080 | IHC | 1:1000 | abcam |
| Goat Anti-Mouse IgG H&L (Alexa Fluor® 488) |  | IHC | 1:1000 | abcam |
|  | ab150113 |  |  |  |
|  |  |  |  |  |
| Cleaved PARP-1 | R380169 | Western Blotting | 1:1000 | Zenbio |
| Cleaved Caspase-3 | R23942 |  | 1:1000 |  |

**Supplementary Table 4**

**List of qRT-PCR primers**

| Primers |  | Sequence（5’→3’） |
| --- | --- | --- |
| LY6H | Forward | GACCACCAACTCCAGCCATT |
|  | Reverse | GATCCTTCCTGCTGCTGCT |
| 18S | Forward | AGAAACGGCTACCACATCCA |
|  | Reverse | CACCAGACTTGCCCTCCA |
| ATG3 | Forward | GCCGTTAAAGAGATCACACTGGAA |
|  | Reverse | TTCACCGCCAGCATCAGTTT |
| ATG5 | Forward | GCTTCGAGATGTGTGGTTTGGA |
|  | Reverse | TGGATAATGCCATTTCAGTGGTG |
| LC3B | Forward | GCCGCACCTTCGAACAAAGA |
|  | Reverse | TGGTGTGGAGACGCTGACC |
| Beclin1 | Forward | GAGGGATGGAAGGGTCTA |
|  | Reverse | GCCTGGGCTGTGGTAAGT |
| PI3K | Forward | CAGTCCTCCCCACCTGATGT |
|  | Reverse | GCGGTAGTGAGATTCGCTGT |
| AKT | Forward | ACCCAACACCTTTGTCATACG |
|  | Reverse | ACTTCCATCTCCTCAGTCGTG |
| mTOR | Forward | CCCAGCTGCTGGAACAAAAA |
|  | Reverse | GTCATGCCCACGTTCCTTAAC |
| AMPK | Forward | TTTGCGTGTACGAAGGAAGAAT |
|  | Reverse | CTCTGTGGAGTAGCAGTCCCT |
