# Supplementary material for: Targeting the LY6H-PI3K/AKT autophagy axis suppresses HCC malignancy and reveals a druggable vulnerability
Source: Cell Death Dis. 2026 Jun 15;17(1):748. doi: 10.1038/s41419-026-08947-z (PMC13493903; doi:10.1038/s41419-026-08947-z)
Supplement: Supplementary file 2 [file 41419_2026_8947_MOESM3_ESM.docx]

**Original western blots for**

**Targeting the LY6H-PI3K/AKT Autophagy Axis Suppresses HCC Malignancy and Reveals a Druggable Vulnerability**

**Figure 1. E**

**Figure 2. D. E**

**Figure 3. C. D. G. H**

**Figure 4. A. F. G**

**Figure 6. G. H**

**Figure S. 1. B. D. F**

**Figure S3. C. D**

**Figure S5. C. D**

**Figure S** **6. D. H. L**

**Figure S** **8I**
